# Spatiotemporal miRNA and transcriptomic network dynamically regulate the developmental and senescence processes of poplar leaves

**DOI:** 10.1093/hr/uhad186

**Published:** 2023-09-26

**Authors:** Kang Du, Shenxiu Jiang, Hao Chen, Yufei Xia, Ruihua Guo, Aoyu Ling, Ting Liao, Wenqi Wu, Xiangyang Kang

**Affiliations:** State Key Laboratory of Tree Genetics and Breeding, National Engineering Research Center of Tree Breeding and Ecological Restoration, College of Biological Sciences and Biotechnology, Beijing Forestry University, Beijing 100083, China; State Key Laboratory of Tree Genetics and Breeding, National Engineering Research Center of Tree Breeding and Ecological Restoration, College of Biological Sciences and Biotechnology, Beijing Forestry University, Beijing 100083, China; State Key Laboratory of Tree Genetics and Breeding, National Engineering Research Center of Tree Breeding and Ecological Restoration, College of Biological Sciences and Biotechnology, Beijing Forestry University, Beijing 100083, China; State Key Laboratory of Tree Genetics and Breeding, National Engineering Research Center of Tree Breeding and Ecological Restoration, College of Biological Sciences and Biotechnology, Beijing Forestry University, Beijing 100083, China; State Key Laboratory of Tree Genetics and Breeding, National Engineering Research Center of Tree Breeding and Ecological Restoration, College of Biological Sciences and Biotechnology, Beijing Forestry University, Beijing 100083, China; State Key Laboratory of Tree Genetics and Breeding, National Engineering Research Center of Tree Breeding and Ecological Restoration, College of Biological Sciences and Biotechnology, Beijing Forestry University, Beijing 100083, China; Institute of Forestry and Pomology, Beijing Academy of Agriculture and Forestry Sciences, Beijing 100093, China; Institute of Crop Sciences, Chinese Academy of Agricultural Sciences, Beijing 100081, China; State Key Laboratory of Tree Genetics and Breeding, National Engineering Research Center of Tree Breeding and Ecological Restoration, College of Biological Sciences and Biotechnology, Beijing Forestry University, Beijing 100083, China

## Abstract

Poplar is an important afforestation and urban greening species. Poplar leaf development occurs in stages, from young to mature and then from mature to senescent; these are accompanied by various phenotypic and physiological changes. However, the associated transcriptional regulatory network is relatively unexplored. We first used principal component analysis to classify poplar leaves at different leaf positions into two stages: developmental maturity (the stage of maximum photosynthetic capacity); and the stage when photosynthetic capacity started to decline and gradually changed to senescence. The two stages were then further subdivided into five intervals by gene expression clustering analysis: young leaves, the period of cell genesis and functional differentiation (L1); young leaves, the period of development and initial formation of photosynthetic capacity (L3–L7); the period of maximum photosynthetic capacity of functional leaves (L9–L13); the period of decreasing photosynthetic capacity of functional leaves (L15–L27); and the period of senescent leaves (L29). Using a weighted co-expression gene network analysis of regulatory genes, high-resolution spatiotemporal transcriptional regulatory networks were constructed to reveal the core regulators that regulate leaf development. Spatiotemporal transcriptome data of poplar leaves revealed dynamic changes in genes and miRNAs during leaf development and identified several core regulators of leaf development, such as GRF5 and MYB5. This in-depth analysis of transcriptional regulation during leaf development provides a theoretical basis for exploring the biological basis of the transcriptional regulation of leaf development and the molecular design of breeding for delaying leaf senescence.

## Introduction

Poplar is one of the world’s most widely distributed, fast-growing, and important tree species that can provide wood quickly [1, 2]. It is also a commonly planted tree species that makes up the landscaping around us [3, 4]. The speed of biomass accumulation in poplar depends on the photosynthesis rate [5]. Landscapes are also closely related to leaf life cycle activities. The life cycle of the leaf involves many important biological processes, including cell division, differentiation and growth, and chlorophyll metabolism. Analysis of the mechanisms regulating leaf growth and development is essential.

The leaf life cycle is usually studied in two stages, i.e. the transitions from young leaf to maturity (Y–M) and from maturity to senescence (M–S) [6]. The process of plant leaf development includes the formation and differentiation of leaf origin meristem, and the whole process of final development from leaf origin to mature leaf [7] includes a very complex physiological and biochemical process that is regulated by the expression of numerous functional genes and transcription factors. In *Arabidopsis*, the regulatory networks involved in leaf development have been analyzed by time series transcriptome analysis [8]. Leaf development is modulated by a multilayered regulatory network that coordinates gene expression, including the negative regulation of target genes by miRNAs and the direct action of transcription factors on downstream genes [9, 10]. The regulatory network is mainly involved in regulating cell growth, division, and differentiation during the young to mature stages of leaf development. Examples include miRNA396, which negatively regulates the GRF (growth-regulating factor) transcription factor family, and miRNA393, which targets ARF1/2/3 (auxin response factor1, 2, 3), affecting leaf size and morphology [11–13]. In poplars, GRF transcription factors are mainly expressed in young tissues and promote cell growth and division through auxin and cytokinin responses, contributing to increased leaf area and photosynthesis rate [14]. *PagKNAT2/6b* could directly activate the expression of *PagBOP1/2a* through CTCTT binding elements, affecting leaf cell axial growth [15].

The senescence phase of the leaf is also accompanied by the active degradation of cellular components and the transfer of nutrient species to nascent sites. Further regulatory mechanisms are involved in the transition from maturation to senescence [16]. The senescence phase of the leaf is also accompanied by the active degradation of cellular components and the transfer of nutrient species to nascent sites [17]. Highly coordinated leaf senescence is the result of the combined action of many senescence-associated genes, known as SAGs [18]. In the leaves of *Populus tomentosa* 3459 autumn senescence-associated genes were identified, and weighted gene co-expression network analysis (WGCNA) identified 115 senescence-associated hub transcription factors in 31 families [19]. Among them, NAC (NAC domain containing protein 69) transcription factors have an important role in regulating leaf senescence in autumn leaves, which is consistent with the findings in *Arabidopsis* [20]. During development, the miR319-*TCP4* (TCP family transcription factor 4) module is involved in regulating cell proliferation, and increased expression of *TCP4* decreases the number of leaf cells [21]. Although these studies elucidated the functions of transcription factors or miRNAs that regulate leaf development in poplars, most were limited to the functional validation of single genes [22, 23]. Consequently, little is known about the dynamic regulation of transcriptional and post-transcriptional levels in the leaf life cycle.

In this study, we used high-throughput sequencing technology to perform high-resolution timing analysis of the leaf life cycle of hybrid poplar (*Populus pseudosimonii × P. nigra* ‘Zheyin 3*#*’ and *P. × beijingensis*), and obtained 45 transcriptomes, 24 miRNAs, and degradome sequencing data during leaf development. In total, 9458 differentially expressed genes were dynamically expressed during leaf development by expression clustering analysis, and 1483 miRNA–mRNA pairs (comprising 1196 genes and 128 miRNAs) were identified. This showed that there were changes in gene expression among different developing leaves, which were divided into five intervals based on the expression trend and were typical of the characteristic zones of leaf transition from young to mature to senescence. The gene dynamic regulatory network of the poplar leaf life cycle was constructed using WGCNA. Several transcription factors, including GRF, NAC, MYB (myb domain protein 15), and other gene families, were identified as core regulators affecting leaf growth and development. Therefore, we constructed a dynamic regulatory network of the poplar leaf life cycle transcriptome to analyze the core regulators affecting leaf growth and development, which provides a useful reference for further understanding the gene expression patterns of leaf development and their dominant regulators.

## Results

### Transcriptome clustering and phenotypic changes during leaf development in *Populus*

**Figure 1:**
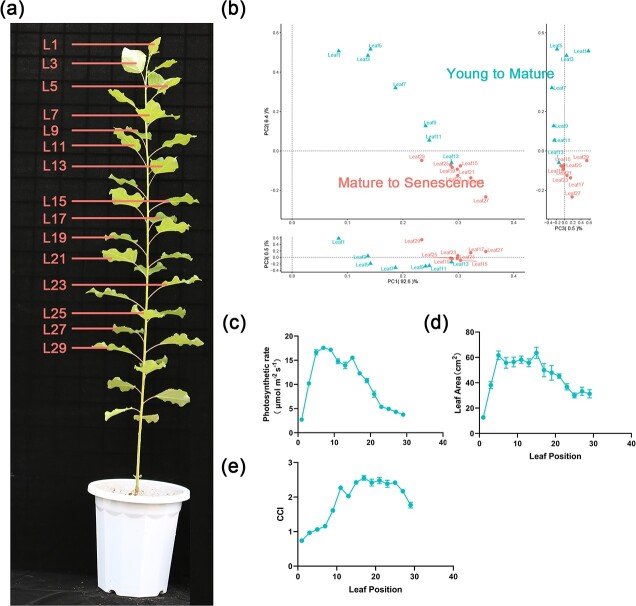
Sample collection and cluster analysis of the poplar leaf transcriptome and miRNAs **a** Fifteen leaf positions were collected for RNA-seq, and seven leaf positions were collected for small RNA sequencing. **b** 3D PCA of the transcriptomes of all 15 sampling points. **c**–**e** Phenotype (photosynthesis rate, leaf area, content of chlorophyll) of poplar leaf at the Y–M stage and M–S stage.

Time-series transcriptome sequencing and small RNA sequencing were performed on leaves at different developmental stages, with three biological replicates per time point, on 45 transcriptome samples and 24 small RNA samples (Fig. 1a). The average matching rate of all transcriptome samples was ~83.1% and gene expression was normalized by FPKM (fragments per kilobase of transcript per million fragments mapped) using *Populus trichocarpa* as the reference genome (Supplementary Data Tables S1 and S2). Gene expression in samples obtained at different leaf developmental stages was analyzed; overall gene expression conformed to a normal distribution (Supplementary Data Fig. S1a, Supplementary Data Table S3). Subsequently, genes with expression >.5 in all samples were selected for further analysis.

The leaf transcriptome can be broadly classified into two taxa (Fig. 1b, Supplementary Data Fig. S1b). Seven time points from L1 to L15 were grouped into one category, with L13 as an outlier, while L17 and later leaves were grouped into another category. This differentiation between the leaf developmental stages was done to better analyze the regulatory mechanisms of the leaf developmental process. Leaf phenotypes have shown that photosynthesis and leaf area peaked at L5 and remained high with leaf development, and started to show a slow decrease after L15 (Fig. 1c and d). Chlorophyll content reached its highest level at L13–L15 and started to decrease at L27 as the leaf developed (Fig. 1e). Thus, L1–L13 was defined as the young to mature stage and L15–L29 as the mature to senescent stage. The stage differentiation of leaf development can help us to more effectively resolve the regular changes in gene expression and its core regulators during leaf development.

### Dynamic changes in transcription factor genes are critical for leaf development

**Figure 2:**
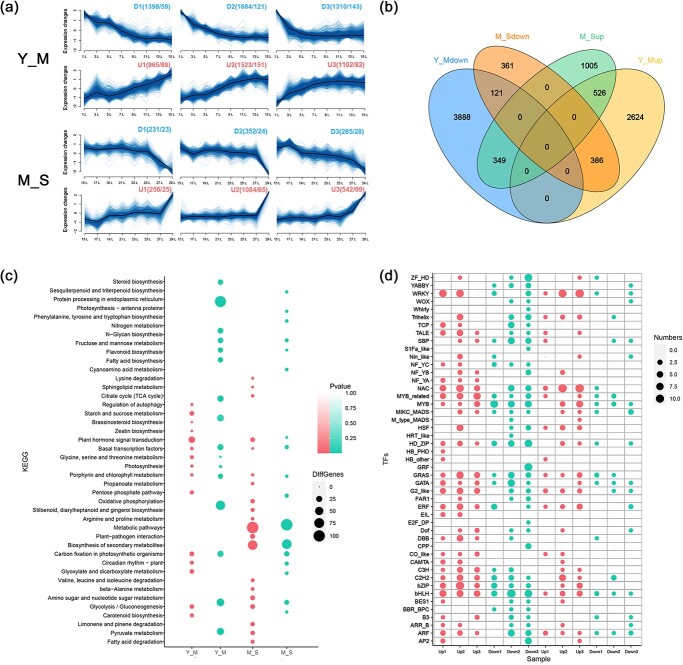
Dynamic changes and functional analysis of genes and transcription factors **a** Fuzzy clustering of gene expression in the Y–M and M–S phases, each divided into increasing (U1, U2, U3) and decreasing (D1, D2, D3) trends. **b** Venn diagram showing the number of genes included in the four clusters at two stages. **c** KEGG functional enrichment of four categories of genes from the Y–M and M–S stages. The *P* value represents the degree of pathway enrichment. **d** Number of transcription factors in different families of 12 clusters at two stages.

Transcriptome data from the two leaf developmental stages (Y–M, M–S) were clustered using the Mfuzz package [24]. In total, 9878 differentially expressed genes were identified, and each stage was classified into three expression trends as upregulated (U1, U2, U3) or downregulated (D1, D2, D3) based on expression (Supplementary Data Tables S4 and S5). In the Y–M leaf development stage, 4392 genes, including 323 transcription factors, gradually decreased with leaf development, and 3590 genes, including 321 transcription factors, gradually increased with leaf development. During the leaf transition from maturation to senescence, 868 genes gradually decreased with leaf development, including 75 transcription factors, and 1882 genes gradually increased with leaf development, including 159 transcription factors (Fig. 2a). Analysis using a Venn diagram showed that there were 677 gene expression regulation across all stages of leaf development; notably, 121 differentially expressed genes consistently tended to be decreased with leaf development, whereas 526 differentially expressed genes tended to increase with leaf development (Fig. 2b).

KEGG (Kyoto Encyclopedia of Genes and Genomes) pathway enrichment analysis was performed on differentially expressed genes in the two developmental stages (Fig. 2c). Genes involved in plant hormone signal transduction, porphyrin and chlorophyll metabolism, glycolysis/glycogenesis, and carbon fixation in photosynthetic organisms revealed significant differentially expressed genes in both stages (Y–M, M–S). Genes related to photosynthesis, starch and sucrose metabolism, zeatin biosynthesis, and brassinosteroid biosynthesis pathways were differentially expressed mainly in the Y–M stage. The metabolic pathways, fatty acid degradation, and other pathways were mainly differentially expressed during the transition from maturity to senescence.

### Analysis of expression patterns of genes regulated by transcription factors during the lifespan

To further investigate how differentially expressed genes function in a coordinated manner during leaf development, we further detailed the tendency of transcription factors to change during leaf developmental stages. In total, 2136 transcription factors were identified in the poplar, of which 878 were significantly differentially expressed at different stages of development. These transcription factors belonged to 47 families (Fig. 2d). There were 644 differentially expressed transcription factors in the Y–M stage and 234 differentially expressed transcription factors in the M–S stage. During leaf development stages, 23 genes of seven transcription factor families, comprising GRF, E2F/DP, CPP, BBR-BPC, Whirly, EIL, and HB-PHD, were significantly differentially expressed only at the Y–M stage. The expression of five transcription factors was significantly upregulated with leaf development, and 18 transcription factors were significantly downregulated with leaf development. Interestingly, 628 transcription factors from 40 transcription factor families, including WRKY, MYB, NAC, GRAS, GATA, bHLH, and ARF, were significantly differentially expressed at both the Y–M and M–S stages. Among them, 232 transcription factors were significantly upregulated and 214 were significantly downregulated at the Y–M stage; 133 transcription factors were significantly upregulated and 49 were significantly downregulated at the M–S stage.

**Figure 3:**
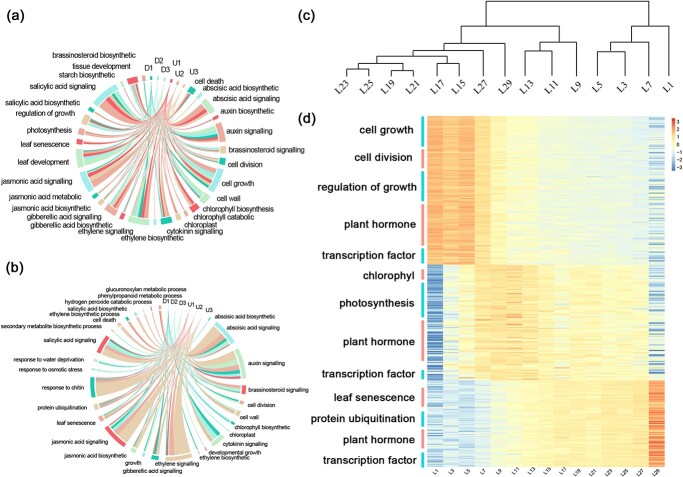
Dynamic expression function of genes in the two stages. **a**, **b** Y–M (**a**) and M–S (**b**) circle maps of transcription factors involved in regulating biological processes. U1–U3 represent transcription factors that increased with leaf position. D1–D3 represent transcription factors that decreased with leaf position. Lines indicate transcription factors involved in the biological process at the end of the line. **c** Cluster dendrogram of the genes from all 15 sampling points. **d** Heat map of gene expression in pathways associated with leaf development.

To further analyze the dynamic regulatory role of transcription factors during leaf development, genes with different expression trends in each of the two stages were annotated with Gene Ontology (GO) functions. In Fig. 3 we link transcription factors belonging to specific GO terms with lines around the circles, to show that these transcription factors are directly involved in the regulation of functional pathways (Fig. 3a and b). In the Y–M stage, 116 transcription factors are mainly involved in the regulation of auxin, cytokinin, cell growth and division, chlorophyll synthesis, and photosynthesis. In the M–S stage, 57 transcription factors are involved in the regulation of hormones, such as abscisic acid (ABA), jasmonic acid (JA), and salicylic acid (SA), as well as genes related to a variety of metabolic pathways, abiotic stress-related pathways, and protein ubiquitination degradation pathways. Thus, transcription factors are dynamically expressed in several ways, such as hormones, cell growth and development, and metabolic processes, and participate in the whole of the processes of the leaf life cycle.

Expression clustering analysis was performed for 173 transcription factors and 323 functional genes obtained from functional enrichment analysis (Fig. 3c). Leaves at 15 positions were divided into four taxonomic groups based on gene expression, where leaves at L1 were classified as group I; L3, L5 and L7 were classified as group II; L9, L11, and L13 were classified as group III; and L15 and beyond were classified as group IV. Unlike principal component analysis (Fig. 1b), expression clustering analysis classified L13 as leaf Y–M stage and L15 as leaf M–S stage. This indicates that, for this study material, around L15 should be the point where the photosynthetic rate of functional leaves starts to decrease. With further division of L15–L29, L29 was reclassified as group V and L15–L27 as group VI.

The gene expression heat map (Fig. 3d) displays the developmental status of leaves at different leaf positions and their functions. Among them, 43 structural genes and 40 transcription factors, such as GRF9, GRF5, TCP4, and KNAT7, are involved in four pathways, comprising cell growth, cell division, growth regulation, and phytohormones. The expression of these genes was higher in leaves at L1–L5, with a decreasing pattern since, and very low expression in leaves at L29. In addition, 78 structural genes and 22 transcription factors, such as ANT and GATA9, are involved in chlorophyll, photosynthesis, and phytohormone-related pathways. The expression of these genes was extremely low in L1 and increased significantly thereafter, reaching a maximum in L7–L13 and gradually decreasing in L15–L27 leaves, with even lower expression in L29. Finally, 77 structural genes and 36 transcription factors, including WRKY23, SPL, and MYB5, were enriched in leaf senescence, protein ubiquitination, and hormone-related pathways. The expression of these genes was relatively low in L1–L7, significantly higher in L9–L27, and highest in L29.

### Expression patterns of miRNA during leaf life

**Figure 4:**
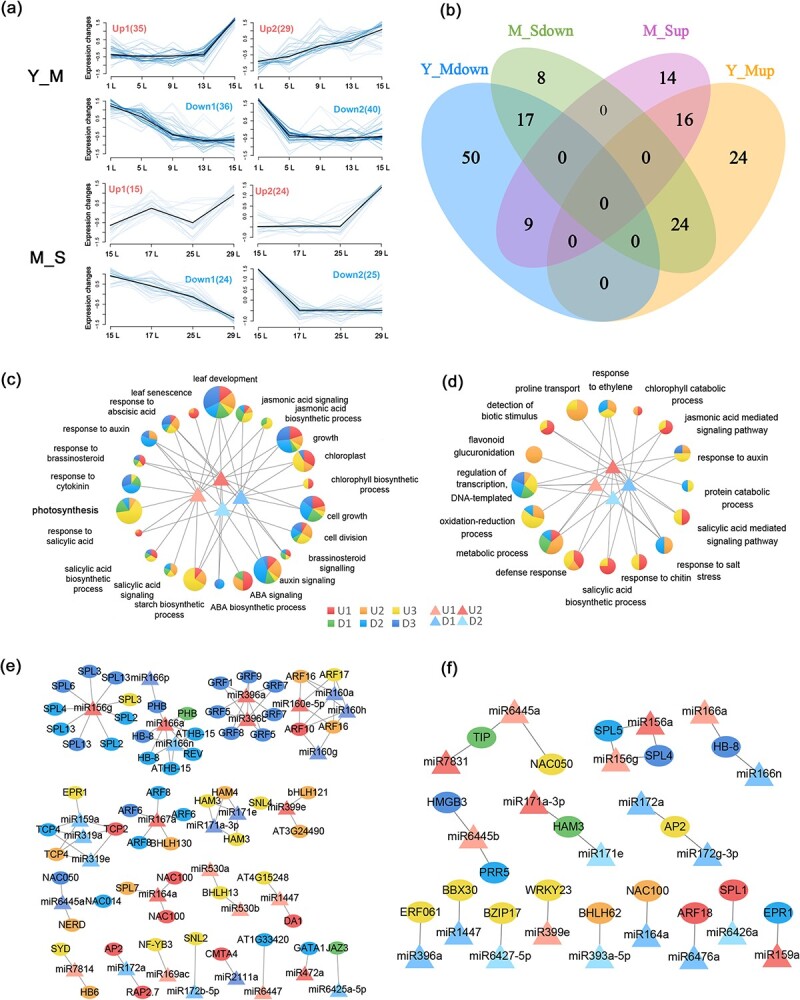
Dynamic changes and target analysis of miRNA. **a** Fuzzy clustering of miRNA expression in the Y–M and M–S phases, each divided into increasing (U1, U2) and decreasing (D1, D2) trends. **b** Venn diagram showing the number of miRNAs included in the four clusters at two stages. **c**, **d** KEGG functional enrichment of four categories of miRNA target genes from the Y–M (**c**) and M–S (**d**) stages. **e** TFs and their target miRNAs regulatory network at Y-M stage. **f** TFs and their target miRNAs regulatory network at M-S stage.

During plant leaf formation and development, miRNAs are involved in several biological processes through post-transcriptional regulation. In this study, the expression of miRNAs was determined by small RNA sequencing at different time points in the leaf lifespan (Supplementary Data Table S3). Consistent with the transcriptome analysis method, cluster analysis of the miRNA expression pattern (Fig. 4a) was also conducted using Mfuzz; the pattern was divided into two stages (Y–M and M–S), including up- and downregulated miRNAs. Among them, 76 miRNAs gradually decreased with the position of the leaf and 64 miRNAs gradually increased in the Y–M stage. Forty-nine miRNAs gradually decreased with leaf position and 39 miRNAs gradually increased in the leaf M–S stage (Fig. 4a). The number of differentially expressed miRNAs was higher in the Y–M stage than in the M–S stage of leaf development. Venn diagram analysis also showed that there were more differentially expressed miRNAs specific to the Y–M stage than the M–S stage (Fig. 4b). Therefore, miRNA has a more dynamic regulation mechanism in the Y–M stage of leaves, similar to genes.

Using degradome data to correlate the transcriptome and miRNA, we identified 1483 miRNA–mRNA relationships (Supplementary Data Table S6). GO annotation of the target genes revealed that these miRNAs were involved in the regulation of leaf development, chlorophyll synthesis, photosynthesis, cell growth and division, and the regulation of gene expression in several hormone-related pathways in the Y–M stage (Fig. 4c). In the M–S stage, miRNAs are mainly involved in regulating chlorophyll metabolism, abiotic stress response, and various metabolic processes, as well as hormone-related pathways such as JA and SA, which negatively regulate growth (Fig. 4d). There were 73 transcription factors among the target genes of miRNAs (Fig. 4f). The above results indicate that miRNAs are involved in the regulation of leaf development by exerting control of genes that participate in different processes during the leaf lifespan, via different regulatory mechanisms in the Y–M and M–S stages.

### Coexpression network analysis of regulation patterns at different stages of the leaf life cycle

**Figure 5:**
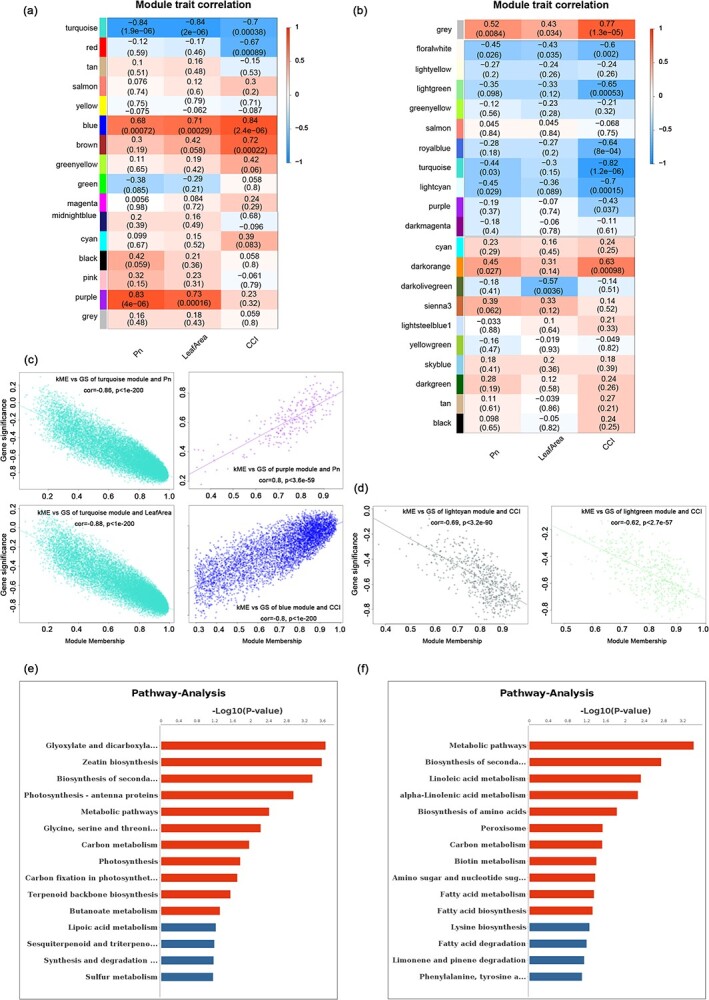
Coexpression network analysis of genes during leaf development, and identification of critical genes. **a**, **b** Module–phenotype association analysis for (**a**) Y–M and (**b**) M–S stages. Each row corresponds to a colored module and each column corresponds to one trait. The correlation coefficient between modules and traits was expressed as *r*. Positive correlation (*r* > 0) is indicated and negative correlation (*r* < 0) is indicated. The correlations and *P*-values (significance tests) between modules and traits are shown in colored boxes. **c**, **d** Scatter plots for Y–M (**c**) and M–S (**d**) showing correlations between genes in modules and phenotypes of every internode. **e**, **f** Genes functionally enriched in the Y–M (**e**) and M–S (**f**) stages by KEGG analysis.

To further analyze the leaf gene expression regulatory network and identify core regulatory factors, we constructed a weighted gene coexpression regulatory network for analysis. As there were significant differences in gene expression patterns between the Y–M and M–S stages, WGCNA was performed separately for both stages (Fig. 5a and b). In Y–M, 16 modules were identified overall, as well as 1 gray module. Gray modules represent the remaining set of genes with no obvious clustering pattern of expression and are generally not considered further. Correlations between modules and leaf photosynthetic rate, leaf area, and chlorophyll content index were calculated (Fig. 5a, b). Gene expression was characterized using module signature genes (ME in Fig. 5). Then, three modules (colored turquoise, purple, and blue in Fig. 5) that were significantly correlated with the phenotype were selected for further analysis. The turquoise module contained 15 923 genes that were significantly negatively correlated with leaf area (*r* = −0.84, *P* = 2.0 × 10^−6^) and photosynthesis rate (*r* = −0.84, *P* = 1.9 × 10^−6^), and presented some negative correlation with chlorophyll content (*r* = −0.7, *P* = 0.00038) (Supplementary Data Fig. S2). To further validate the correlation between genes and phenotypes in the turquoise module, the correlation coefficients between each gene and phenotype were analyzed using scatter plots (Fig. 5c). Genes in the turquoise module were significantly correlated with leaf area (*r* = −0.88, *P* = 1 × 10^−200^), photosynthetic rate (*r* = −0.86, *P* = 1 × 10^−200^), and chlorophyll content (*r* = −0.72, *P* = 1 × 10^−200^). The temporal dynamics revealed that the expression of these genes decreased gradually with leaf development. Functional enrichment analysis of the genes in the turquoise module revealed that these genes are involved mainly in zeatin biosynthesis, biosynthesis of secondary metabolites, photosynthesis, photosynthesis-antenna proteins, and carbon metabolism-related pathways (Fig. 5e, Supplementary Data Table S7).

The blue module contained 5202 genes that were significantly positively correlated with the photosynthetic rate (*r* = 0.68, *P* = 0.00072), leaf area (*r* = 0.71, *P* = 0.00029), and chlorophyll content (*r* = 0.84, *P* = 2.4e × 10^−6^). Scatter analysis revealed a high correlation between genes in the blue module and chlorophyll content (*r* = 0.8, *P* = 1 × 10^−200^), and a slightly lower correlation with photosynthesis rate (*r* = 0.55, *P* = 1 × 10^−200^) and leaf area (*r* = 0.66, *P* = 1 × 10^−200^). Functional enrichment analysis revealed that genes in the blue module were mainly involved in carotenoid biosynthesis, carbon fixation in photosynthetic organisms, carbon metabolism, and plant hormone signal transduction (Supplementary Data Table S7). The purple module contained 260 genes that were positively correlated with photosynthesis rate (*r* = 0.83, *P* = 4 × 10^−6^) and leaf area (*r* = 0.73, *P* = 0.00016). Scatter plot analysis revealed significant positive correlations between genes in the purple module and both photosynthesis rate (*r* = 0.8, *P* = 3.6 × 10^−59^) and leaf area (*r* = 0.71, *P* = 3.5 × 10^−41^). Functional enrichment analysis showed that genes in the purple module genes were concentrated in pathways related to photosynthesis, photosynthesis-antenna proteins, zeatin biosynthesis, carbon fixation in photosynthetic organisms, and metabolic pathways (Supplementary Data Table S7). Thus, the genes contained in the three modules are involved in the regulation of leaf growth and development in the Y–M stage.

In the M–S stage, gene coexpression network analysis grouped expressed genes into 29 modules for association analysis of phenotypic traits from maturity to senescence (Supplementary Data Fig. S2). We found that several modules, including light green (*r* = −0.65, *P* = 0.00053), turquoise (*r* = −0.82, *P* = 1.2 × 10^−6^), and light cyan (*r* = −0.7, *P* = 0.00015), were significantly associated with chlorophyll content. However, after scatter plot analysis of genes and chlorophyll content within modules, only the light cyan (*r* = −0.69, *P* = 3.2 × 10^−90^) and light green (*r* = −0.62, *P* = 2.7 × 10^−57^) modules were significantly correlated (*r* > 0.5 or *r* < −0.5) (Fig. 5d). Functional analysis of the genes in these two modules indicates that they are involved in a variety of metabolic pathways, particularly porphyrin and chlorophyll metabolism (Fig. 5f, Supplementary Data Table S7). Therefore, we suggest that the genes in these two modules play a major regulatory role in the M–S stage.

### Discovery and functional analysis of key genes

**Figure 6:**
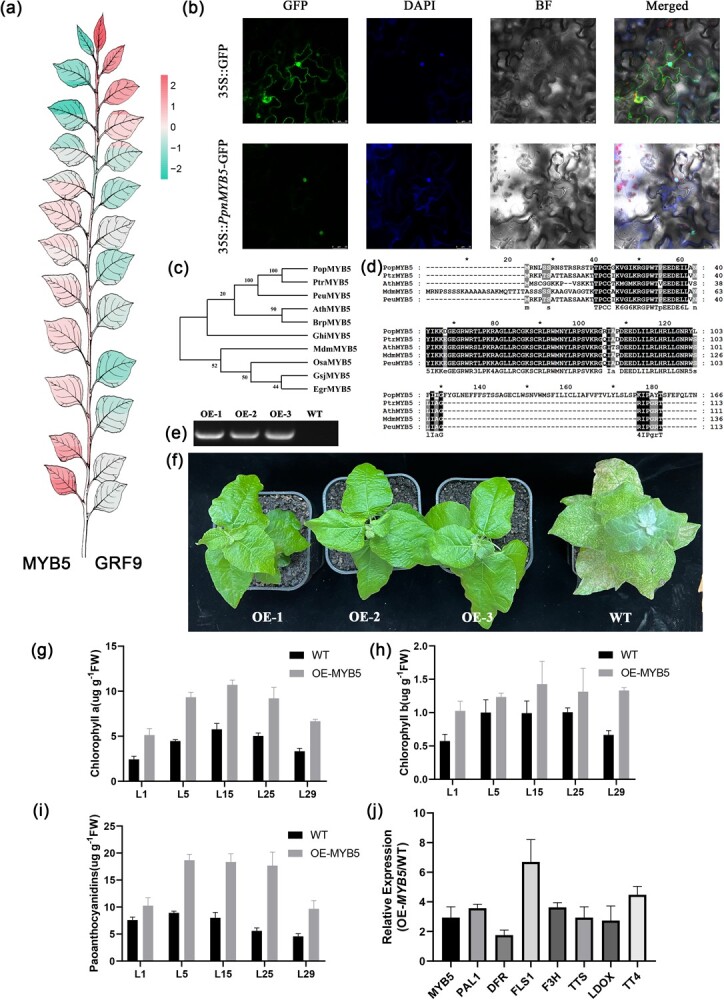
Role of core transcription factors in the regulation of leaf development. **a** Heat map of GRF9 and MYB5 in leaf development. **b** Subcellular localization analysis of MYB5. **c**, **d** Coding sequence and protein sequence analysis of MYB5. **e** Identification of overexpression of MYB5 by PCR. **f** Phenotype of OEMYB5. **g**–**i** Quantitative determination of content of chlorophyll a, chlorophyll b, and proanthocyanidin in PpnMyb5-OE plants. **j** Quantitative reverse transcription–PCR analysis of MYB5 and PAL1, DFR, FLS1, F3H, TT8, LDOX, and TT4 genes in the wild type.

Based on the significance of genes in the modules and the module gene connectivity, a gene coexpression regulatory network was constructed (Fig. 5a and b). The top-ranked transcription factors in each module were selected as the core regulators (Supplementary Data Table S8). In the Y–M stage, several GRF transcription factor genes are present in the turquoise module, e.g. GRF9 and GRF12. The expression of these GRFs decreased gradually with leaf development (Fig. 6a) and was classified as cluster D3 in the Y–M stage. The turquoise module also includes cytokinin and growth hormone metabolism genes, including *IPT5* (isopentenyltransferase 5), *CKX3* (cytokinin oxidase 3), *IAA19* (indole-3-acetic acid inducible 9), and *AAE16* (AMP-dependent synthetase and ligase family protein), and several *CYCD* (cyclin D-type protein) and *XTH* (xyloglucan endotransglucosylase/hydrolase) genes encoding cell elongation and growth and cyclic division proteins. In the M–S stage, transcription factors such as *MYB5* and *WRKY4* in the light cyan module act as core regulators. The expression of transcription factors gradually decreased as the leaves developed from maturity to senescence. Among them, the *MYB5* transcription factor belongs to cluster U3, whereas the expression pattern of the *WRKY4* transcription factor belongs to cluster U1.

**Figure 7:**
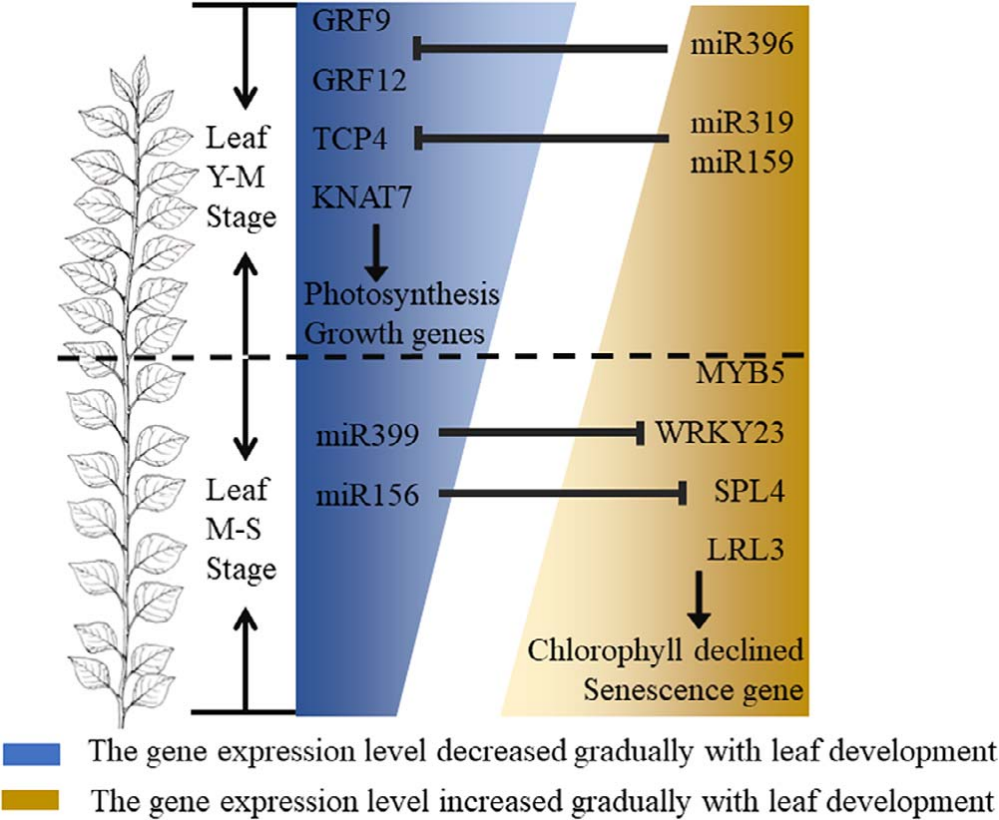
Models proposed for leaf development.

The subcellular localization of *PpnMYB5* has been identified as the nucleus (Fig. 6b). A phylogenetic tree analysis of protein sequences revealed that *PpnMYB5* in P. *pseudosimonii × P. nigra* ‘Zheyin 3#’ (Ppn) has high homology to *P. trichocarpa* (Fig. 6c). Transcription factor *PpnMYB5* has a functional structural domain specific to MYB transcription factors, suggesting that *MYB5* transcription factors may have the same function in different species (Fig. 6d). *PpnMYB*5 overexpression increased leaf total chlorophyll, proanthocyanidins, carotenoids, and chlorophyll a and b content compared with the wild type by promoting the expression of structural genes (Fig. 6e–i). These results confirm that *PpnMYB5*, a core regulator appearing in the light cyan module, is involved in regulating leaf development.

## Discussion

As a photosynthetic organ, the leaf plays a crucial role in the accumulation of biomass [25]. Plant leaves undergo a series of physiological and morphological transitions during the young–mature–senescent life cycle, during which the regulation of gene and miRNA expression is tightly integrated to ensure the correct development of leaves [6, 26]. Studies have shown that transcription factor families are involved in regulating leaf developmental processes, e.g. TCP, KONX, bHLH, GRF, NAC, and other families [27–29]. TCP14 and TCP15 can co-modulate leaf size in *Arabidopsis* [30], KNAT1 and KNAT2 regulate leaf margin development [31], and bHLH (basic helix–loop–helix family) gene LMI-like silencing leads to leaf widening and also works with KNOX to regulate leaf development [32]. In this study, the dynamics of gene expression during the poplar leaf lifespan was analyzed to explore how miRNAs and genes are regulated during the two stages of leaf transition (Y–M and M–S). This study has provided an important molecular basis for breeding by molecular design, which is expected to slow down leaf senescence and improve photosynthetic assimilation capacity of leaves.

Gene expression during leaf development shows a temporal pattern [7]. In *Arabidopsis*, leaf development was studied in two stages: young–mature (4–16 days) and mature–senescent (18–30 days) [6, 10]. The transcriptome changes in senescent leaves showed more coordinated temporal changes and more complex regulatory networks than the young stage. Based on the expression patterns associated with leaf phenotypes and dynamic transcriptome data, poplar leaf development can be classified into two stages: young to mature (L1–L15) and mature to senescent (L17–L29). Functional enrichment analysis and expression clustering analysis of leaf gene expression allowed the division of the above two phases into five distinctly variable intervals with distinct gene expression trends. Among them, L1 is a separate interval, which is in the stage of cell division and functional differentiation. The expression of genes related to cell division, growth, growth hormones, cytokinin, and growth-regulating transcription factors was at its highest level in this leaf position (Fig. 2c). L3–L7 were grouped into an interval in which the expression of genes related to cell division and growth gradually decreased, but still maintained a high level relative to the leaves after 15 leaves. Expression of genes involved in chlorophyll synthesis and photosynthesis began to increase gradually. The leaves in this interval have basically completed morphological establishment, and photosynthetic capacity has started to be enhanced, which is the period for the initial formation of young leaf development and photosynthetic capacity. L9–L13 were grouped into an interval in which the genes highly expressed in L1 had dropped to a lower level, and the expression of photosynthesis-related genes reached the highest level. The leaves in this interval are fully mature and the phase of strongest photosynthetic capacity of functional leaves. L15–L27 were grouped into an interval where the expression of photosynthesis-related genes decreased slightly, while the expression of genes related to leaf senescence and protein ubiquitination began to show an increasing tendency. At this stage, the photosynthetic capacity of functional leaves started to decrease. At L29, photosynthesis-related genes dropped to the lowest level, while leaf senescence-related regulatory genes reached the highest level. At this leaf position, photosynthetic capacity was lost, marking the beginning of the leaf senescence period. It should be noted that there should be some variation in the position and number of leaves included in the relevant five-leaf position intervals if the species are different, or even different genotypes of the same species, different culture environments of the same genotype and different sampling times. In poplar, the leaf area after L21 is smaller (Fig. 1d). The reason for this is that the chloroplasts are not fully developed and the photosynthetic rate is low when the leaves are at a young age. A sufficient number of functional leaves is needed to provide nutrients to ensure their full development. In this study, the leaves were formed at about L21, and in order to ensure the maximum leaf area development of young leaves this was only possible when the functional leaves reached about L11.

The effects of miRNAs on transcription factors are particularly important for the regulation of leaf development [33, 34]. For example, miRNA396c regulates leaf growth and development by directly targeting the GRF family of genes [35]. In 84 K poplar, *PagGRF12* suppressed xylem development by regulating the expression of *PagXND1* [36]. *PpnGRF5* significantly increased leaf area by directly repressing the expression of the *CKX1* gene, encoding a cytokinin-degrading enzyme [14]. In poplar, the miRNA156-SPL module was found to affect anthocyanin biosynthesis and negatively regulate leaf area [22]. In this study, 15 transcription factors and structural genes that ranked in the top three core regulatory modules were screened by WGCNA, including eight transcription factors: GRF9, GRF12, KNAT7, TCP4, MYB5, SPL4, LRL3, and WRKY23 (Table 1). The degradome helps us to further identify the post-transcriptional regulatory mechanisms and expand the dimensionality of the regulatory mechanisms of leaf development. Degradome sequencing analysis showed that, besides KNAT7, MYB5, and LRL3, the remaining five transcription factors were also regulated by miRNAs, such as miR396, miR319, miR159, miR156, and miR399. These eight transcription factors showed patterned expression changes with leaf development (Fig. 3d). Among them, transcription factors GRF9, GRF12, KNAT7, and TCP4, had the highest expression at L1 and expression gradually decreased with leaf development. It was shown that TCP4 can directly bind to the promoter regions of the PORB and DVR genes, which are involved in regulating leaf chlorophyll content and directly involved in affecting leaf cell proliferation [37, 38]. GRF9 is involved in regulating cell division by regulating the expression of ORG3, which determines the size of the leaf [39]. In addition, KNAT7 can also interact with GRF family genes to regulate leaf cell wall thickness and cell expansion [40]. The transcription factors MYB5, SPL4, LRL3, and WRKY23 had lower expression at L1, and their expression gradually increased with leaf development. In poplars, MYB165 and MYB194 could repress the expression of phenylpropanoid enzyme genes; consequently, the contents of several phenylpropanoids, such as anthocyanins and pro-anthocyanidins, were significantly reduced in overexpressing plants [27].

## Conclusions

In this study, the transcriptional landscape of the poplar leaf life cycle was constructed from a high-resolution leaf transcriptome and miRNA dataset. Gene expression during leaf development is temporal and has a phase transition characteristic between L1, L3–L7, L9–L13, L15–L27, and L29. A regulatory network of gene expression during leaf development and senescence was constructed, and several core transcriptional regulators were identified. In the Y–M stage, we identified eight transcription factors for leaf development, among which GRF9, GRF12, KNAT7, and TCP4 were involved in promoting leaf growth and morphogenesis. These genes are regulated by miR396 and miR159, and gene expression gradually decreases with leaf development. In the M–S phase, MYB5 and SPL4 are involved in regulating leaf cell senescence and chlorophyll degradation, regulated by miR399 and miR156. The expression of these transcription factors gradually increased with leaf development (Fig. 7). The results will help to enhance our understanding of the mechanisms of leaf growth and development and provide an efficient genetic resource for improving the rate of biomass accumulation in poplars.

## Materials and methods

### Plant material, sample collection, and physiological measurements

The offspring populations of *Populus* section *Tacamahaca* (*P. pseudosimonii × P. nigra* ‘Zheyin 3#’, Ppn) were generated in our previous study [40]. Material was grown in a greenhouse (16 h light/8 h dark) at Beijing Forestry University. After 4 months of growth, the photosynthesis rate of whole plant leaves was measured by leaf order using a photosynthesis meter (LiC-6400, LiCor, Inc., Lincoln, NE, USA). The chlorophyll content index and leaf area were measured using a CCM-200 Plus chlorophyll content meter (Opti-Sciences Inc., USA) and a CI-203 portable laser leaf area meter. Leaf samples were collected from leaf positions 1, 3, 5, 7, 9, 11, 13, 15, 17, 19, 21, 23, 25, 27, and 29 of the poplars. Every three plants were combined to make one biological sample, and three biological replicates were collected at each leaf position. The samples were stored in liquid nitrogen at low temperatures and used to extract RNA for transcriptome sequencing and miRNA sequencing for library construction.

### Small RNA and RNA-seq data analysis

Total RNA from leaves was extracted using the TRIzol Universal Kit (DP424 Tiangen, Beijing, China). Quality control of the extracted RNA was performed using 1% agarose gels and a NanoPhotometer N80 (Implen, Germany). RNA-seq libraries were generated with a HiPure Plant RNA Mini Kit (Magen, China) in accordance with the manufacturer’s protocols. Small RNA libraries were generated using an NEB Next Illumina^®^ (NEB, USA.) and sent to Novogene for sequencing as well as RNA-seq library creation. The raw RNA-seq data were filtered to remove low-quality bases and adapters by Trimmomatic, and the filtered clean data were mapped to the *Populus* 3.1 reference genome by HISAT2 (v. 2.20). After processing the raw data, gene expression was presented following FPKM normalization, and miRNA expression was presented following TPM (transcripts per million) normalization. Functional annotation was performed using the Genomewide GO and KEGG databases.

### Expression clustering and weighted gene coexpression network analysis

The hierarchical structure analysis of the samples was used with the online website tool imageGP (https://www.bic.ac.cn/ImageGP/) based on Pearson’s correlation. Principal component analysis was performed using the ‘prcomp’ function in R (v. 3.6.1). The R package ‘Mfuzz’ was used to cluster dynamically expressed genes and miRNAs based on their expression profiles at different leaf positions. Genes from the Y–M and M–S stages were filtered for FPKM >0.1, and gene coexpression networks were constructed via the WGCNA package using the default settings of R. The gene regulatory networks were visualized using Cytoscape v. 3.6.0 (http://www.cytoscape.org/).

### Phylogenetic analysis

The phylogenetic analysis used the *MYB5* protein sequences from *Populus*, *Oryza sativa*, and *Arabidopsis thaliana*. Protein sequence alignment was performed with default parameters; then, the resulting sequence was subjected to the maximum likelihood method PhyML to generate a phylogenetic tree in MEGA v. 7.0 [41].

### Genetic transformation and subcellular localization

The full-length coding sequence of *PpnMYB5* was cloned and transferred into the pBI121-eGFP vector. The fusion construct (35S::*MYB5*-GFP) and control construct (35S::GFP) vectors were transiently expressed in tobacco leaves. The construct (35S::*MYB5*-GFP) was introduced into *Agrobacterium* strain GV3101, and then transformed into poplar 84 K cultures via the leaf disk method [42]. The leaf disks were washed with double-distilled water and cultured on medium for callus inducement supplemented with 500 mg/l cefotaxime and 50 mg/l kanamycin for 10–30 days in the dark. Shoot and root regeneration from the calli was induced on medium supplemented with 100 mg/l kanamycin for several weeks to months.

### qPCR validation

Template strands for qRT–PCR were conducted using the FastKing cDNA Kit (KR116, Tiangen, Beijing, China). The 7500 Fast system was used for the PCR process as well as for fluorescence signal analysis, with three technical replicates conducted per gene. *Populus* actin was used as an endogenous reference gene. All primers are listed in Supplementary Data Table S9. The 2(−ΔΔCt) method was used to calculate transcription levels [43, 44].

### Determination of leaf chlorophyll and pigment content

Total flavonoid was extracted with buffer (60% ethanol, 0.1 mol/l hydrochloric acid ethanol), and determined by spectrophotometry (T6 New Century, Beijing, China). Proanthocyanidin content was determined by the method of Prior [14], adding 500 μl PA solution (30% acetic acid, 70% acetone) to 0.2 g of ground poplar leaves, ultrasonic extraction in an ice bath for 30 min, centrifugation at 2000 g for 10 min at 15°C, and taking the supernatant. The obtained supernatant was extracted twice with trichloromethane for the chlorophyll and aqueous phases. Proanthocyanidin (CAS:4852-22-6) was used as the standard and 80% ethanol solution was used as the blank control, and the absorbance at 640 nm was measured using an enzyme marker and converted to proanthocyanidin content [45].

### Statistical analysis

Statistical analyses of gene expression and physiological measures were performed with the GraphPrism software. For wild type versus overexpression comparison, *P* values were derived from Student’s *t* test. For multigroup comparisons, *P* values were derived from ANOVA; *P* < .05 was considered significant (*) and *P* < .01 was considered highly significant (**).

## Supplementary Material

Web_Material_uhad186
