# Supplementary figures and images for: Spatiotemporal miRNA and transcriptomic network dynamically regulate the developmental and senescence processes of poplar leaves
Source: Hortic Res. 2023 Sep 26;10(10):uhad186. doi: 10.1093/hr/uhad186 (PMC10611553; doi:10.1093/hr/uhad186)

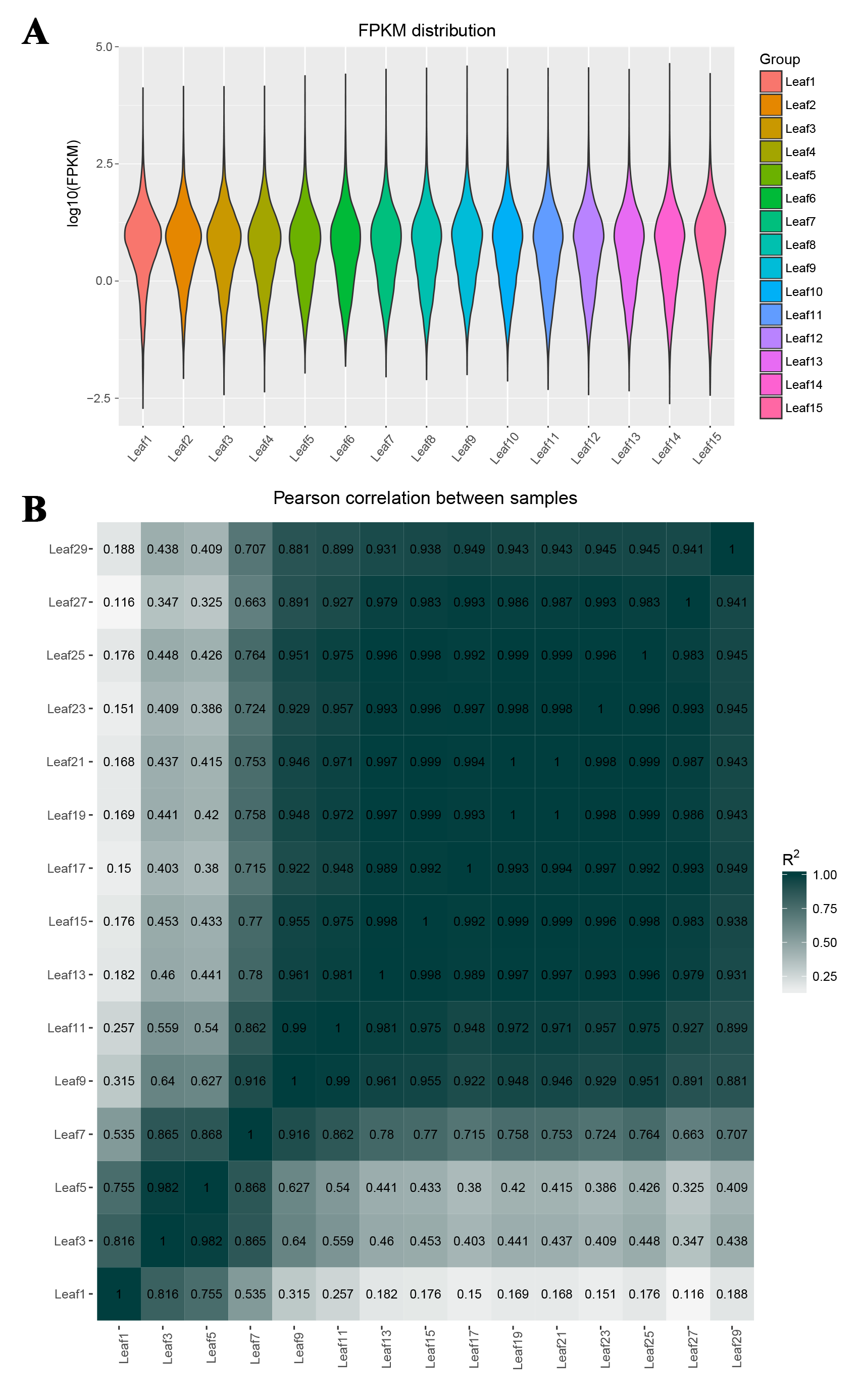

Supplement: Web_Material_uhad186 [file web_material_uhad186.zip › Fig.S1.tif]

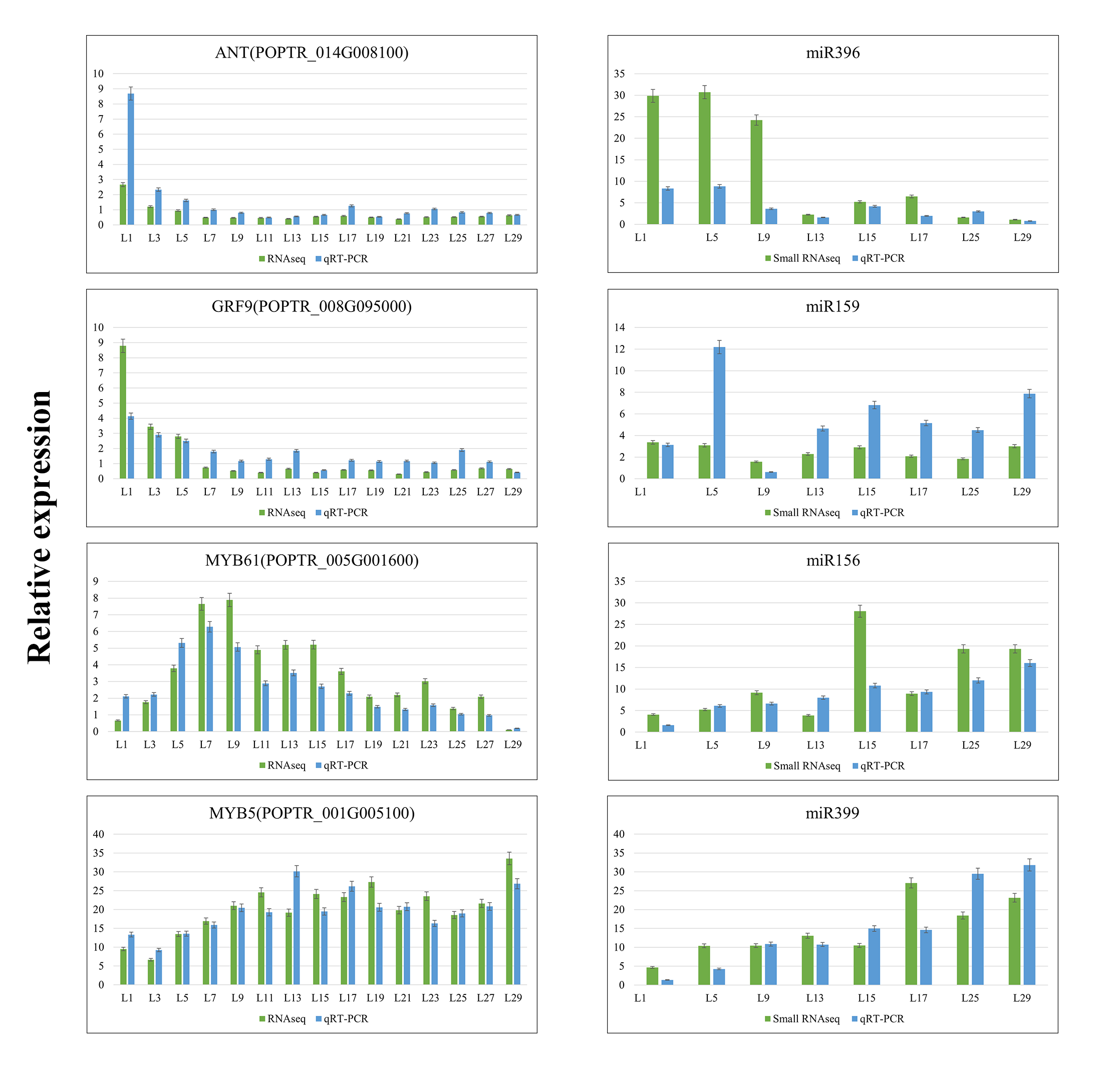

Supplement: Web_Material_uhad186 [file web_material_uhad186.zip › Fig.S2.tif]

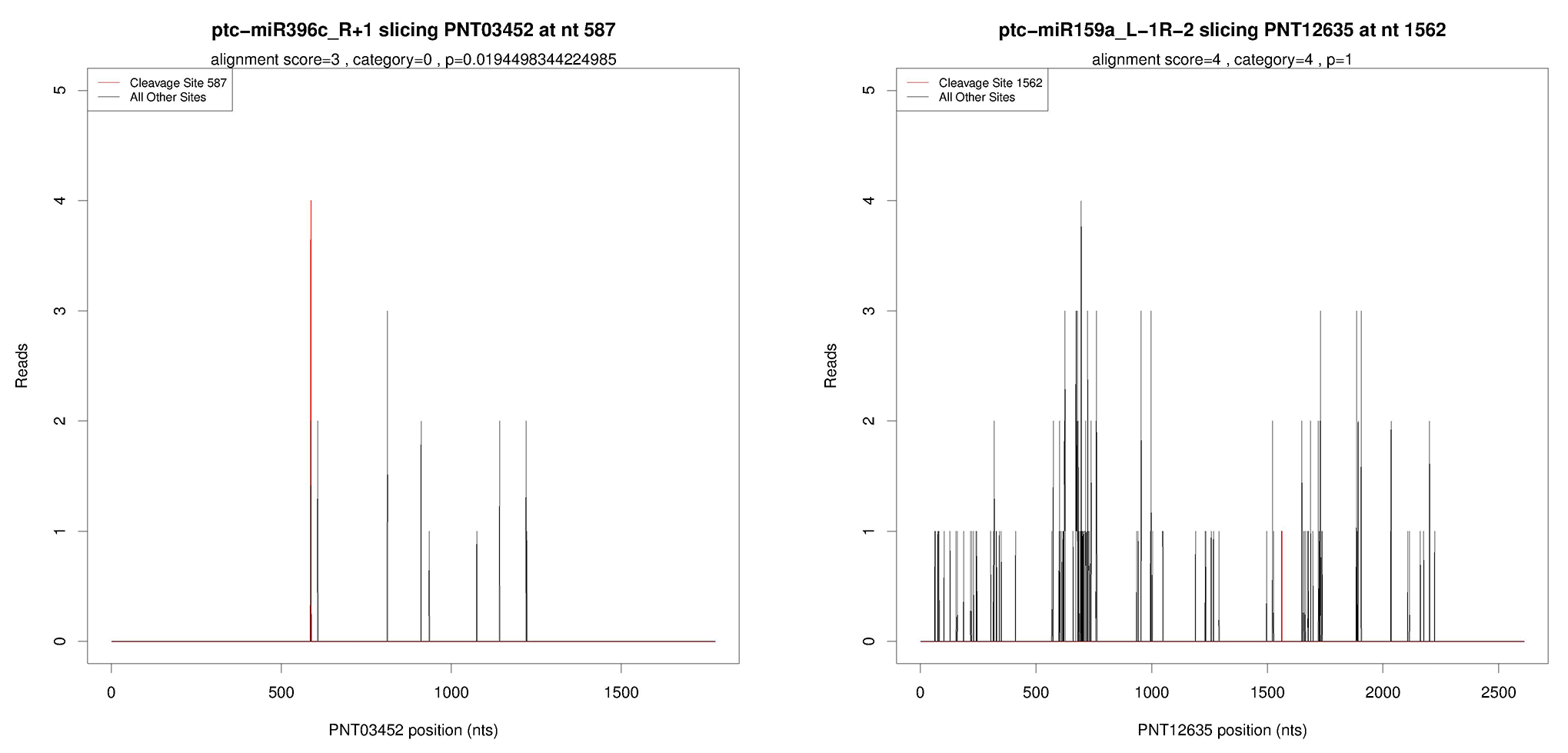

Supplement: Web_Material_uhad186 [file web_material_uhad186.zip › Fig.S3.tif]

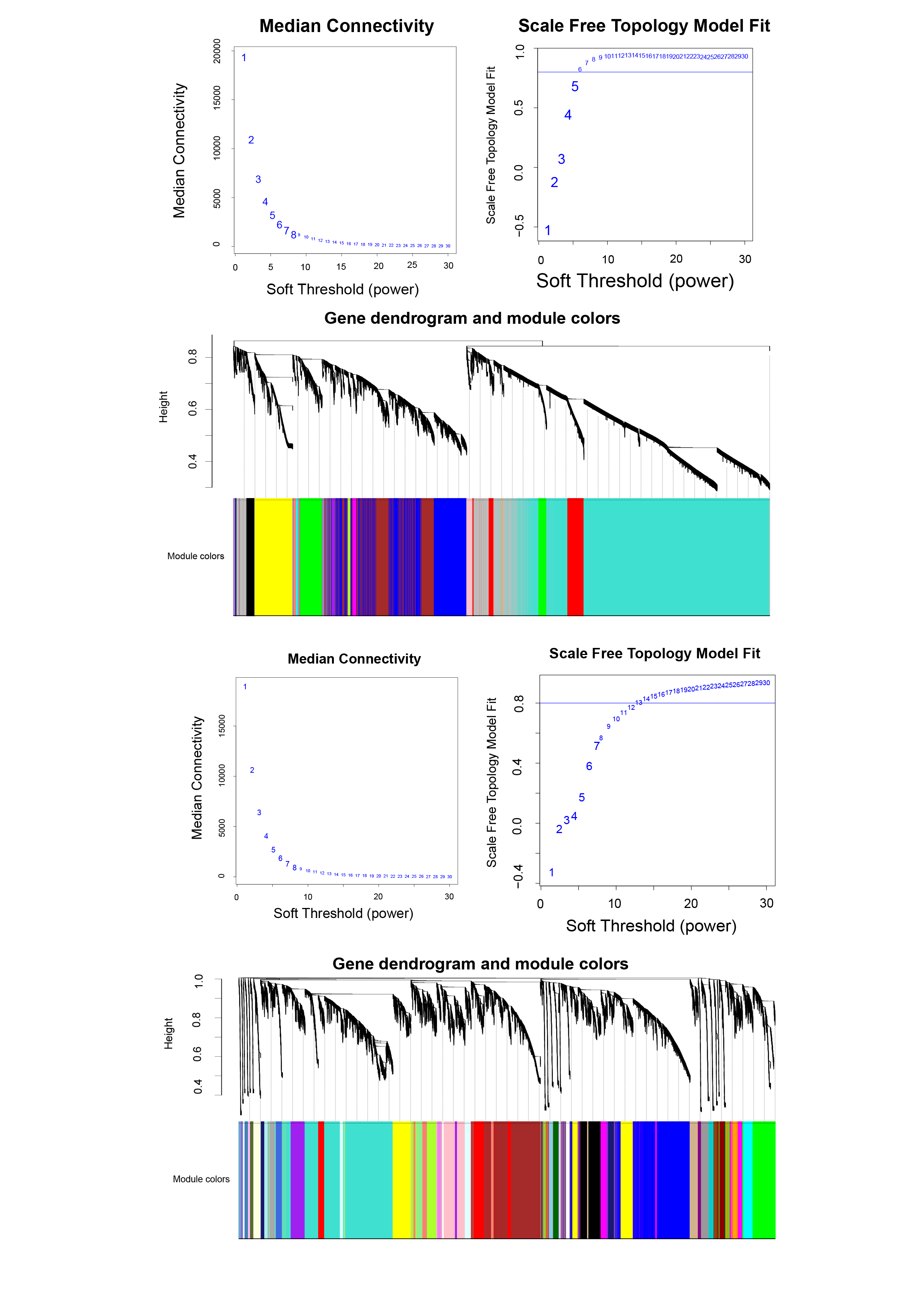

Supplement: Web_Material_uhad186 [file web_material_uhad186.zip › Fig.S4.tif]
